# Exploring the Potential of *Genista ulicina* Phytochemicals as Natural Biocontrol Agents: A Comparative In Vitro and In Silico Analysis

**DOI:** 10.3390/toxins17090452

**Published:** 2025-09-06

**Authors:** Roukia Zatout, Ouided Benslama, Fatima Zohra Makhlouf, Alessio Cimmino, Maria Michela Salvatore, Anna Andolfi, Radhia Manel Kolla, Marco Masi

**Affiliations:** 1 Department of Microbial Biotechnology, Faculty of Natural and Life Science, University of Blida 1, Ouled Yaich 09000, Algeria; 2 Department of Natural and Life Sciences, Faculty of Exact Sciences and Natural and Life Sciences, Larbi Ben M’Hidi University, Oum El Bouaghi 04000, Algeria; 3 Higher National School of Biotechnology Taoufik Khaznadar, Nouveau Pôle Universitaire Ali Mendjeli, BP. E66, Constantine 25100, Algeria; 4 Department of Chemical Sciences, University of Naples Federico II, 80126 Naples, Italy; 5 Department of Veterinary Medicine and Animal Production, University of Naples Federico II, 80137 Naples, Italy

**Keywords:** *Genista ulicina*, molecular identification, phytochemical profiling, antifungal activity, phytotoxicity, docking molecular, biopesticides, sustainable agriculture

## Abstract

Development of new sustainable pesticides represents a real challenge for researchers due to environmental issues and public health aspects. In fact, the overuse of chemical pesticides has led to environmental damage, loss of biodiversity, and pesticide-resistant pests. In a framework characterized by the necessity of new sustainable agricultural practices, this study investigates the plant *Genista ulicina* as a producer of bioactive compounds for potential application as eco-friendly biopesticides. First, both roots and aerial parts of *G. ulicina* were extracted and the main compounds in the crude extracts were identified via GC-MS. Subsequently, the crude extracts were submitted to antifungal and phytotoxic assays. In particular, the antifungal effects were evaluated on three common phytopathogenic fungi, *Fusarium oxysporum*, *Alternaria alternata*, and *Botrytis cinerea*, while phytotoxic activity was evaluated on two weed species: *Euphorbia peplus* L. and *Oxalis corniculata* L. Further insights were obtained on the herbicidal potential of phytochemical compounds produced by *G. ulicina* through in silico investigations. In particular, molecular docking analyses were performed against three key enzymes involved in essential plant metabolic pathways: acetohydroxyacid synthase (AHAS), 4-hydroxyphenylpyruvate dioxygenase (HPPD), and protoporphyrinogen oxidase (PPO). Among the compounds identified, linolelaidic acid methyl ester, 1-monolinolein, stearic acid, and palmitic acid derivatives showed promising binding affinities and favorable interaction patterns compared to reference ligands. Selected phytochemicals from *G. ulicina* show potential as inhibitors of key herbicide targets, suggesting their value as promising leads in the development of sustainable bio-based weed control agents.

## 1. Introduction

In recent years, the agricultural industry has faced increasing challenges due to the overuse of chemical pesticides. While these agents are effective in pest control, their widespread and often indiscriminate application has caused significant environmental damage, including soil and water contamination. Additionally, they have negatively impacted biodiversity by harming non-target species and contributing to the emergence of pesticide-resistant pest populations [1,2].

In response, increasing attention has been directed toward more sustainable and environmentally friendly pest management strategies. Among these, plant-based extracts have gained prominence owing to their rich content of bioactive compounds [3,4]. These natural compounds often exhibit antimicrobial, insecticidal, and fungicidal properties, positioning them as promising alternatives to conventional synthetic pesticides [5,6,7]. Unlike conventional chemicals, plant extracts tend to act in an eco-friendly and selective manner and usually pose lower toxicity risks to non-target organisms [8,9]. Furthermore, their complex chemical profiles may reduce the likelihood of resistance development, enhancing their value in integrated pest management (IPM) programs [7]. In parallel, modern herbicide development has focused on targeting specific metabolic pathways unique to plants, thereby offering high selectivity and minimizing impacts on animals and humans [10]. In this context, three key plant enzymes, acetohydroxyacid synthase (AHAS), 4-hydroxyphenylpyruvate dioxygenase (HPPD), and protoporphyrinogen oxidase (PPO), have emerged as valuable targets due to their central roles in plant growth and survival.

Plants offer a diverse and structurally unique source of bioactive compounds with lower environmental impact and toxicity profiles compared to synthetic herbicides [11]. Their structural variety also allows for novel mechanisms of enzyme inhibition, potentially helping to overcome herbicide resistance in weed populations [12].

Within this framework, the chemo-biodiversity of plants, particularly those belonging to the *Genista* genus (Fabaceae), represents a promising reservoir of phytochemical compounds. Several *Genista* species have demonstrated antimicrobial, antioxidant, anti-inflammatory, and allelopathic effects [13,14].

The present study aims to identify *Genista ulicina* and explore the potential of its crude organic extracts as potential natural agents for sustainable agriculture. Gas chromatography–mass spectrometry (GC-MS) analysis of the crude extracts revealed the presence of various compounds, including cinnamic acids and fatty acids. Subsequently, antifungal and phytotoxic bioassays were performed to determine the bioactivity of *G. ulicina* crude extracts. Additionally, molecular docking studies were conducted on identified phytochemicals targeting three key herbicide-related enzymes: acetohydroxyacid synthase (AHAS), 4-hydroxyphenylpyruvate dioxygenase (HPPD), and protoporphyrinogen oxidase (PPO), and their binding affinities and interaction profiles were compared with those of known inhibitors. This integrated approach contributes to the development of effective, environmentally friendly, plant-based biopesticides as alternatives to conventional synthetics.

## 2. Results and Discussion

### 2.1. Identification of the Studied Plant

**Figure 1:**
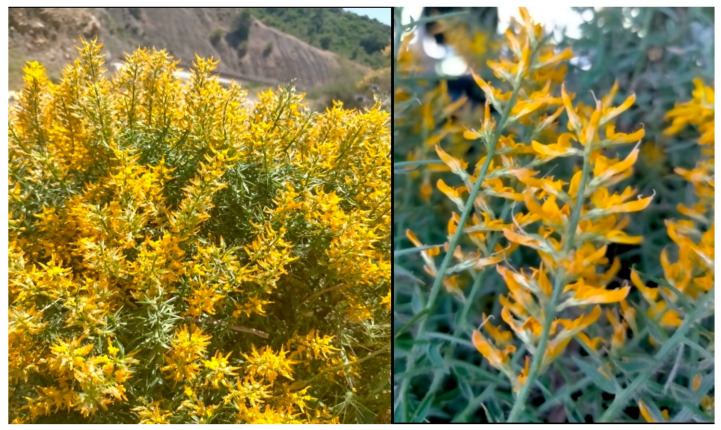
The studied plant *Genista ulicina*.

The identification of *G. ulicina* (Figure 1) was initially based on morphological observations. The plant is characterized by small, narrow, linear to lanceolate leaves and bright yellow papilionaceous flowers. Its stems are rigid, covered with fine hairs, and the plant forms dense thickets due to its bushy and branched growth habit. Reproductive structures include legumes (seed pods), typical of the Fabaceae family, which house several small seeds. The species is widely distributed across the Mediterranean region, particularly in Algeria. It is well adapted to poor, rocky, and dry soils, demonstrating resilience in arid environments [15,16]. To confirm the species identification at a molecular level, DNA was extracted from the plant material, and the internal transcribed spacer (ITS) region of the ribosomal DNA was sequenced. This region is commonly used for plant species identification due to its variability, which makes it suitable for distinguishing closely related species. For molecular confirmation, DNA was extracted from the plant material, and the internal transcribed spacer (ITS) region of the ribosomal DNA was sequenced. The resulting ITS sequence was compared to the GenBank database using the Basic Local Alignment Search Tool (BLAST). The comparison confirmed the species identity as *G. ulicina*, with the sequence matching the reference strain deposited under GenBank accession number PQ118722 [17].

### 2.2. Extraction and Chemical Characterization

Aerial parts and roots of *G. ulicina* were extracted as detailed in Section 4 (Materials and Methods), to obtain two different extracts (*n*-hexane and dichloromethane). These were analyzed via TLC, NMR and GC-MS. The TLC profile showed the presence of lipophilic metabolites in the *n*-hexane extract and a more complex mixture of low-molecular-weight compounds in DCM (Figure S1). ^1^H NMR spectra of the four organic extracts (Figures S2–S5) showed signals belonging to different classes of natural products. Crude extracts were analyzed via GC-MS after derivatization with *N,O*-bis(trimethylsilyl)trifluoroacetamide (BSTFA) (Figures S6 and S7). Table 1 shows the complete list of compounds identified in *n*-hexane and dichloromethane extracts of the aerial parts and roots of *G. ulicina*.

### 2.3. Antifungal Activity

The antifungal activity of *Genista ulicina* extracts was evaluated against three important phytopathogenic fungi: *Fusarium oxysporum*, *Alternaria alternata*, and *Botrytis cinerea* [18,19]. The results revealed both aerial and root extracts exhibited strong inhibitory activity against the tested phytopathogens (Table 2). Statistical analysis confirmed significant differences in inhibition percentages across treatments (*p* < 0.05), except against *B. cinerea*, where all extracts achieved complete inhibition (100%).

For *F. oxysporum*, both aerial extracts (n-hexane and DCM) displayed complete inhibition (100%), whereas root extracts showed slightly lower but still very high inhibition (96.4–94.7%). In the case of *A. alternata*, root extracts (*n*-hexane and DCM) demonstrated the highest inhibition (97–98%), significantly higher than aerial extracts (87% and 67%). By contrast, *B. cinerea* was fully inhibited (100%) by all extracts, regardless of plant part or solvent. No statistical differences were observed, confirming a broad-spectrum fungicidal activity of *G. ulicina* against this pathogen.

This is especially significant considering the agricultural impact of *F. oxysporum* and *A. alternata*, which are known to cause considerable crop losses. The efficacy of these extracts highlights their potential as natural alternatives to synthetic fungicides in plant disease management. Additionally, the total growth suppression of *B. cinerea* suggests a potent and possibly more specific mode of action against this fungus, potentially affecting critical functions such as cell wall synthesis or mitosis. The differential antifungal activity observed across fungal species may be attributed to structural and biochemical variations among the pathogens. Differences in cell wall composition, enzyme systems, and susceptibility to phytochemicals influence how each species responds to plant-derived compounds. Moreover, the variation in efficacy between aerial and root extracts of *G. ulicina* suggests a diverse phytochemical profile, with each part containing unique secondary metabolites that may exert distinct antifungal mechanisms [20].

### 2.4. Phytotoxic Activity

The phytotoxic effects of *n*-hexane and dichloromethane (DCM) extracts, derived from both the aerial and root parts of *G. ulicina,* were evaluated on *Oxalis corniculata* and *Euphorbia peplus* by leaf puncture assay. A two-way ANOVA was conducted to assess the effects of plant part and extract concentration on necrotic area formation in the target plant.

#### 2.4.1. Phytotoxic Activity on *Oxalis corniculata*

A two-way ANOVA was performed to evaluate the effects of plant part (aerial and root) and extract concentration (0.5, 1.0, 2.0 mg/mL, and control) on the necrotic area induced by *G. ulicina n*-hexane and dichloromethane (DCM) extracts when tested on *O. corniculata* (Table 3).

Our findings showed that both plant parts and concentrations had significant effects on necrotic area. In particular, extract concentration showed a highly significant effect, with all extract types producing progressively larger necrotic areas as the concentration increased. The highest necrotic activity was observed at 2.0 mg/mL for all treatments, while the control group exhibited the lowest effects. Post hoc comparisons (Tukey’s HSD) confirmed that root extracts induced significantly greater necrosis than aerial parts across all concentrations, with the most pronounced observed for root hexane extract at 2.0 mg/mL (55.30 ± 0.75 mm^2^). All extract treatments exhibited significantly larger necrotic areas than the control. Moreover, the significant interaction between plant part and concentration indicates that the increase in necrosis with concentration was more pronounced in root extracts.

#### 2.4.2. Phytotoxic Activity on *Euphorbia peplus*

The phytotoxic activity on *E. peplus* was evaluated by measuring necrotic area formation on target plant using *n*-hexane and dichloromethane (DCM) extracts from both aerial and root parts of *G. ulicina* at increasing concentrations (0.5, 1.0, and 2.0 mg/mL), and compared to a control (4% MeOH) (Table 4).

**Figure 2:**
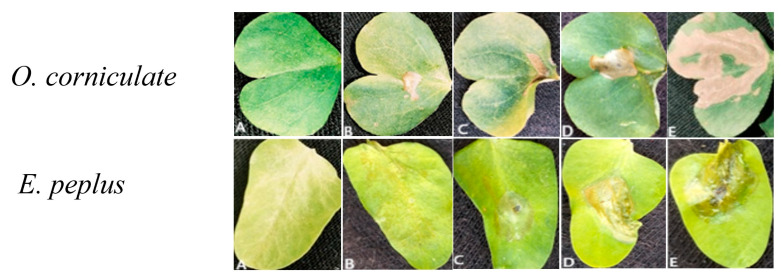
Necrotic area induced by *G. ulicina n*-hexane and DCM extracts at 2 mg/mL on *O. corniculate* and on *E. peplus*: (**A**) methanol (MeOH 4%), (**B**) DCM extract of the aerial part, (**C**) *n*-hexane extract of the aerial part, (**D**) DCM extract of the root, (**E**) *n*-hexane extract of the root.

Two-way ANOVA indicated a clear concentration-dependent increase in necrotic area for both *n*-hexane and DCM extracts. At all concentrations, sample extracts exhibited significantly greater necrotic areas compared to the control, which consistently showed negligible values. This confirms that the solvent (4% methanol) had no inherent phytotoxic effect. A significant effect of both plant part and concentration was observed (*p* ≤ 0.05), with the most substantial necrotic areas recorded at the highest concentration (2.0 mg/mL). Post hoc analysis (Tukey’s HSD) showed that root extracts induced significantly larger necrotic areas than aerial extracts at most concentrations, particularly in DCM extracts. For *n*-hexane extracts, although necrosis values were higher in root samples compared to aerial parts, the difference was not statistically significant. The greatest phytotoxic effect was observed with the root *n*-hexane extract at 2.0 mg/mL (26.57 ± 0.57 mm^2^), closely followed by root DCM at the same concentration (25.40 ± 0.41 mm^2^). Both *O. corniculata* and *E. peplus* extracts showed significant, concentration-dependent phytotoxic effects, with root extracts generally causing more necrosis than aerial parts (Figure 2).

This suggests that roots may contain higher levels of allelopathic compounds [21,22]. In *O. corniculata*, the root *n*-hexane extract at 2.0 mg/mL showed the strongest activity, indicating a richness in non-polar phytotoxins. Similarly, *E. peplus* root extracts, especially the *n*-hexane and DCM ones, induced greater necrosis than aerial parts, though the difference was more pronounced in DCM extracts. These findings highlight the role of plant part and solvent polarity in phytotoxicity and support the potential of both species as sources of natural herbicidal compounds [23,24].

### 2.5. Molecular Docking Analyses

Acetohydroxyacid synthase (AHAS), also known as acetolactate synthase (ALS), catalyzes the first common step in the biosynthesis of branched-chain essential amino acids—valine, leucine, and isoleucine. This enzyme is a well-established target of several classes of commercial herbicides, including sulfonylureas and imidazolinones, which act by inhibiting its activity and thereby disrupting amino acid synthesis [25,26]. Another crucial enzyme, 4-hydroxyphenylpyruvate dioxygenase (HPPD), is involved in tyrosine catabolism and plastoquinone biosynthesis, processes that are essential for carotenoid production and the protection of photosynthesis. HPPD is the target of widely used herbicides such as mesotrione and isoxaflutole [27,28]. Protoporphyrinogen oxidase (PPO) plays a central role in chlorophyll and heme biosynthesis. Inhibition of PPO leads to the accumulation of phototoxic intermediates, causing oxidative damage, membrane disruption, and eventually plant death [29].

Despite their high efficacy, many synthetic herbicides suffer from significant drawbacks, including persistence in the environment, potential toxicity to non-target species such as aquatic organisms, and health risks to humans upon exposure [30,31,32]. These concerns underscore the urgent need for safer, environmentally friendly alternatives, particularly those derived from natural sources.

**Figure 3:**
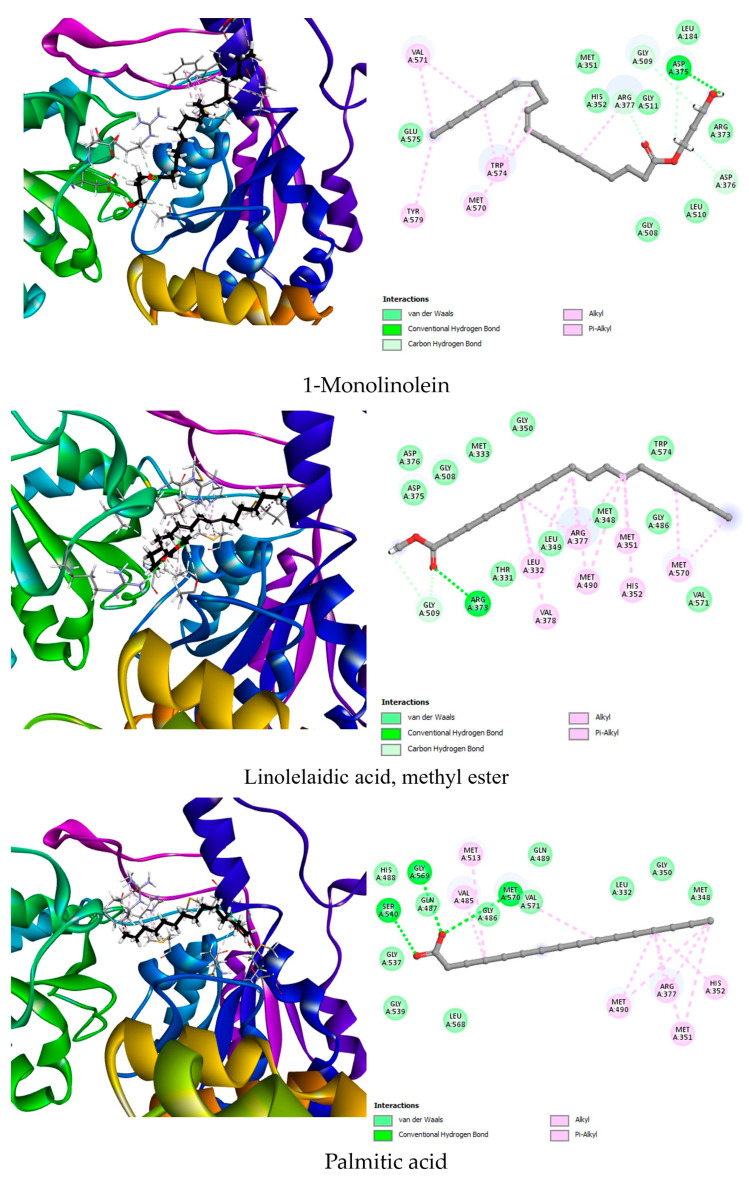
Two-dimensional and 3D representations of the top three phytochemical ligands docked against acetohydroxyacid synthase (AHAS, 1YHZ).

**Figure 4:**
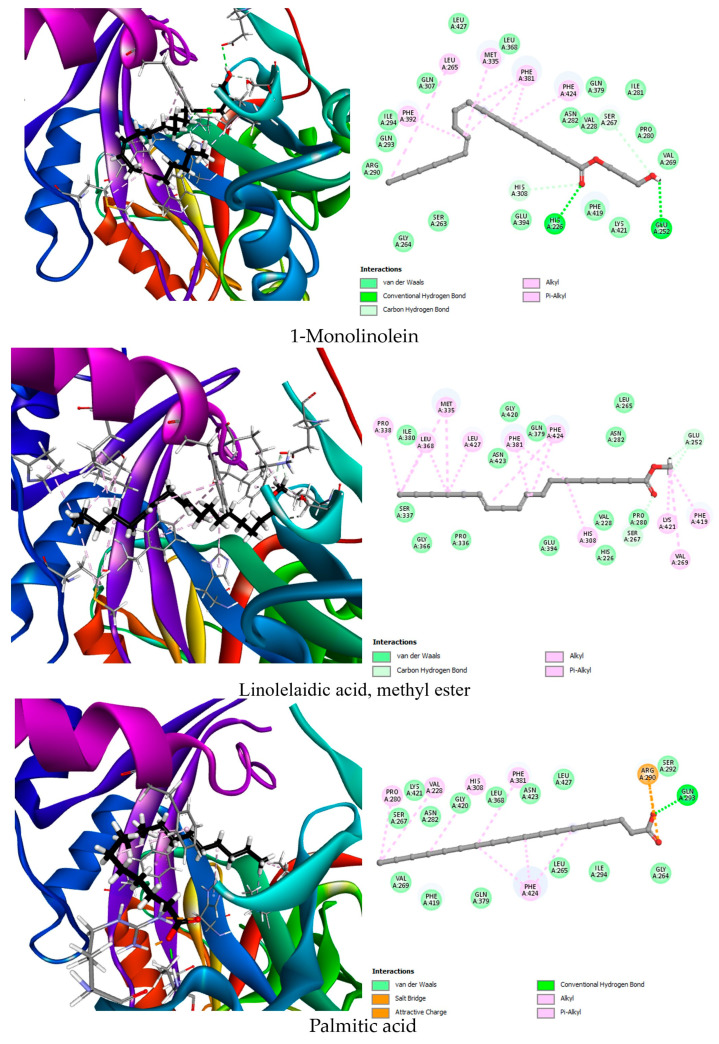
Two-dimensional and 3D representations of the top three phytochemical ligands docked against 4-hydroxyphenylpyruvate dioxygenase (HPPD, 6J63).

**Figure 5:**
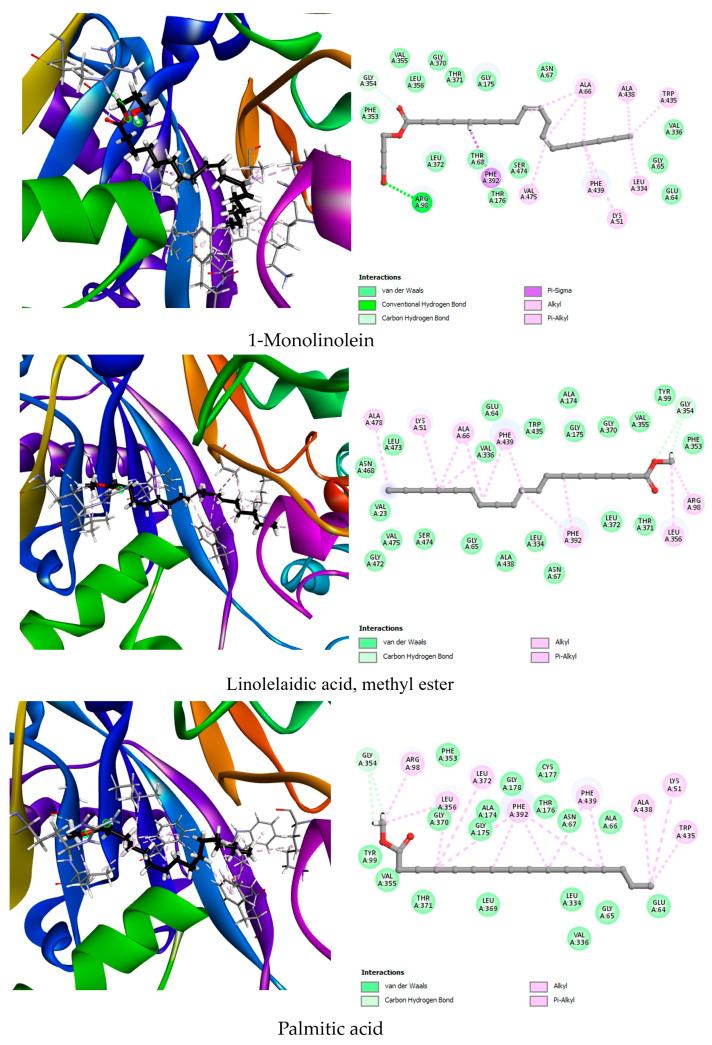
Two-dimensional and 3D representations of the top three phytochemical ligands docked against protoporphyrinogen oxidase (PPO: 1SEZ).

To explore such alternatives, we conducted molecular docking analyses to evaluate the herbicidal potential of 38 phytochemical compounds identified from *G. ulicina* root extracts. Protein targets (AHAS, PDB: 1YHZ; HPPD, PDB: 6J63; PPO, PDB: 1SEZ) were selected based on literature reports highlighting their essential roles in plant metabolism and their established vulnerability to chemical inhibition. Ligands were chosen from GC-MS profiles of *n*-hexane and dichloromethane root extracts, taking into account their relative abundance and biological relevance. The docking scores, hydrogen bond interactions, and hydrophobic contacts between the ligands and each target enzyme are summarized in Table 5 and visually represented in Figure 3, Figure 4 and Figure 5. In addition, a redocking of the co-crystallized ligands was performed to validate the docking protocol and establish baseline binding affinities for comparison. These data were used to highlight the top three phytochemicals for each enzyme based on binding energy and interaction quality.

For AHAS, the co-crystallized ligand 1CS demonstrated a strong binding affinity of −8.5 kcal/mol, forming multiple hydrogen bonds with key active site residues such as Gly508, Asp375, Arg373, and Arg377. Additionally, hydrophobic interactions with Trp574 and Met570 helped stabilize the ligand within the binding pocket. Among the phytochemicals tested, linolelaidic acid methyl ester showed the highest binding affinity (−7.6 kcal/mol), forming hydrogen bonds with Gly509 and Arg373, and interacting hydrophobically with Met351, Met490, Met570, and other residues involved in the catalytic site. This interaction profile suggests a good fit and potential inhibition of AHAS activity. 1-Monolinolein and palmitic acid also exhibited significant binding affinities of −6.8 and −6.7 kcal/mol, respectively. 1-Monolinolein engaged in multiple hydrogen bonds with Asp375, Gly509, and Arg377, key residues involved in substrate stabilization. Its numerous hydrophobic contacts with Trp574, Met570, and Arg377 reinforce its binding. Palmitic acid, although slightly lower in affinity, interacted favorably with Ser540, Gly569, and Met570, again engaging the active core of the enzyme (Figure 3). These findings suggest that these lipophilic molecules can mimic interactions made by commercial AHAS inhibitors.

In the case of HPPD, the native ligand NDT displayed a moderate binding energy of −6.9 kcal/mol, interacting with His226, His308, and Lys421 via hydrogen bonding, and engaging in hydrophobic interactions with Phe424. Interestingly, all top three phytochemicals outperformed the co-crystallized ligand in terms of binding affinity. 1-Monolinolein exhibited the strongest binding affinity (−7.7 kcal/mol), forming hydrogen bonds with His226, His308, Glu252, and Ser267, in addition to hydrophobic interactions with aromatic residues such as Phe381, Phe424, and Phe392. This profile indicates deep insertion into the active pocket, with specific anchoring in the metal coordination and substrate-recognition zones. Linolelaidic acid methyl ester and stearic acid followed closely, with −7.5 and −7.3 kcal/mol, respectively. Both compounds formed relevant interactions with residues known to participate in the catalytic cycle, including Glu252 and His308 (Figure 4). These results highlight the potential of these compounds to interfere with HPPD activity, a major target of bleaching herbicides.

The PPO co-crystallized ligand, OMN, showed a binding affinity of −8.1 kcal/mol, forming hydrogen bonds with Arg98 and Gly178 and hydrophobic interactions with Phe392, Leu334, and Leu372. Remarkably, the phytochemical 1-monolinolein exhibited an even higher binding affinity of −8.7 kcal/mol, establishing hydrogen bonds with Gly354 and Arg98. Its extensive hydrophobic network with Trp435, Phe439, Phe392, and Val475 strongly suggests a stable occupation of the catalytic core. Linolelaidic acid methyl ester also showed impressive docking results (−8.4 kcal/mol), engaging in hydrogen bonding with Gly354 and hydrophobic interactions with key residues such as Phe439, Ala66, and Leu356. Similarly, palmitic acid methyl ester matched the native ligand’s energy (−8.1 kcal/mol) and displayed strong interactions with Gly354, Leu372, and Phe392 (Figure 5). These findings suggest that these lipophilic phytochemicals could act as potent inhibitors of PPO by mimicking the structural and interaction patterns of commercial herbicides.

Among the compounds identified through molecular docking, linolelaidic acid methyl ester, 1-monolinolein, stearic acid, and palmitic acid derivatives exhibited the highest binding affinities toward key herbicide-related targets such as AHAS, HPPD, and PPO. These compounds showed stable interactions with active site residues essential for enzymatic function, suggesting their potential to disrupt critical metabolic pathways in plants. Notably, phytochemical profiling revealed that these bioactive fatty acid derivatives are predominantly present in the *n*-hexane extract of the roots of *G. ulicina*. This correlates strongly with the results of the in vitro phytotoxicity assay, in which the root *n*-hexane extract demonstrated the most potent necrotic effect on *O. corniculata*, with necrotic areas increasing in a dose-dependent manner and reaching an average of 55.3 mm^2^ at 2.0 mg/mL. The convergence between the high concentration of active compounds in the root *n*-hexane fraction and their predicted molecular interactions supports the hypothesis that the strong phytotoxic effects observed are due to the synergistic action of these fatty acid derivatives. These findings highlight the root *n*-hexane extract as a promising source of natural herbicidal agents, with its activity likely mediated through multi-target inhibition mechanisms predicted by docking studies.

## 3. Conclusions

This study confirmed the identity of *G. ulicina* through morphological and molecular analysis. Phytochemical profiling revealed a diversity of bioactive compounds, mainly in root *n*-hexane extracts. Among them, GC-MS identified fatty acid derivatives like linolelaidic acid methyl ester and 1-monolinolein. These extracts showed strong antifungal effects against *F. oxysporum, A. alternata*, and *B. cinerea*. Phytotoxicity assays revealed dose-dependent necrosis on *O. corniculata* and *E. peplus.* Root extracts were more potent than aerial parts in both their antifungal and phytotoxic activity. Molecular docking confirmed high binding affinities to AHAS, HPPD, and PPO herbicide targets. Fatty acid derivatives may act through multi-target inhibitory mechanisms. The biological activity aligns with the high concentration of these compounds in root extracts. *G. ulicina* root extracts show strong potential as natural, eco-friendly bioherbicides and antifungals.

## 4. Materials and Methods

### 4.1. General Experimental Procedures

Thin layer chromatography (TLC) was performed on silica gel (Kieselgel 60, F_254_, 0.25 or 0.50 mm; Merck, Darmstadt, Germany) or reverse phase (Whatman, KC18, F_254_, 0.20 mm). The spots were visualized by exposure to UV radiation (254 nm) or by spraying first with 10% H_2_SO_4_ in methanol and then with 5% phosphomolybdic acid in EtOH, followed by heating at 110 °C for 10 min or by exposure to iodine vapors. ^1^H NMR spectra were recorded at 400 on a Bruker (Karlsruhe, Germany) Anova Advance spectrometer. The spectra were recorded in deuterated solvents which were also used as internal standards. Sigma-Aldrich Co. (St. Louis, MO, USA) supplied all the reagents and the solvents.

### 4.2. Plant Material

**Figure 6:**
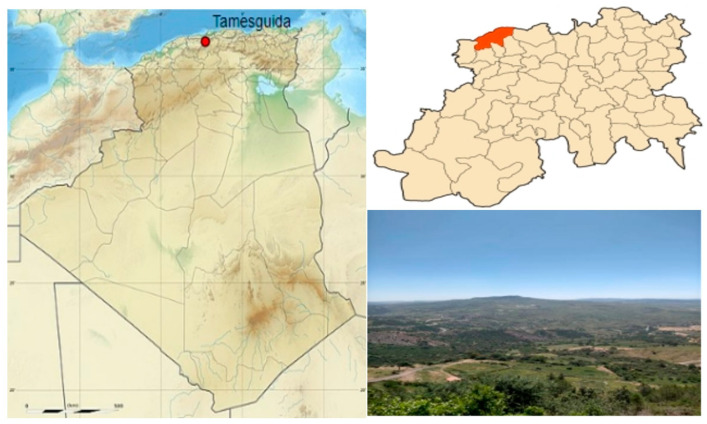
The geographic location of the Tamazguida region.

The plant material was collected during the flowering period in April 2022 by Dr. R. Zatout in the Tamazguida region, which is situated at the bottom of Djebel Mouzaïa that has an altitude of 1604 m, 12 km north-west of Médéa Province (Figure 6) [33]. The collected plants were separated into two parts: aerial and root. These parts were cut into small pieces, spread on a tray, and turned over occasionally. The materials were left to air-dry for two weeks. Once dried, the plant materials were ground into a powder and stored in sealed bags to protect them from moisture until extraction.

### 4.3. Morphological Identification

The morphological identification of the plant involved macroscopic observations and species determination based on key morphological traits such as leaf arrangement, flower morphology, stem characteristics, and pod structure [34]. These traits are essential for distinguishing between closely related species, especially in regions with high floral diversity. Field observations were supplemented with comparisons to taxonomic keys and regional floras to ensure accurate and reliable identification.

### 4.4. Molecular Identification

A voucher specimen of the plant material was identified at ALVALAB, Oviedo 33006, Spain, by Dr. Pablo Alvarado. Total DNA was extracted from dried specimens using a modified protocol based on Murray and Thompson (1980) [35]. PCR amplification was performed for 35 cycles with an annealing temperature of 54 °C (Mullis and Faloona, 1987). The forward primer ITS1F (5′-TCC GTA GGT GAA CCT GCG G-3′) [36] and the reverse primer ITS4 (5′-TCC TCC GCT TAT TGA TAT GC-3′) [37] were used to amplify the ITS rDNA region. Amplicons were sequenced using the PCR primers, and the resulting sequence was deposited in GenBank [38].

### 4.5. Plant Extracts Preparation

Twenty grams of powdered plant material from each part (aerial and roots) was extracted twice by maceration with 140 mL of EtOH:H_2_O (1:1 *v/v*) for 48 h per extraction. The resulting hydroalcoholic extracts from both the aerial and root parts were then subjected to sequential liquid–liquid partitioning using *n*-hexane (3 × 200 mL), followed by DCM (3 × 200 mL). The organic phases were dried over anhydrous Na_2_SO_4_, filtered, and concentrated under reduced pressure to yield the corresponding organic extracts: 15.0 mg (*n*-hexane) and 141.2 mg (DCM) from the aerial parts and 19.3 mg (*n*-hexane) and 197.2 mg (DCM) from the roots. The chromatographic profile of the four extracts has been evaluated by TLC using chloroform-isopropanol 9:1 as eluent (Figure S0). The remaining aqueous phase was lyophilized to obtain the aqueous fraction [39].

### 4.6. GC-MS Analysis

GC-MS data were acquired on the raw crude extracts (*n*-hexane and dichloromethane extracts) of roots and arial parts after trimethylsilylation with *N,O*-bis(trimethylsilyl)trifluoroacetamide (BSTFA) (Fluka, Buchs, Switzerland) as previously reported [40]. GC-MS analyses were conducted with an Agilent 6850 GC (Milan, Italy), equipped with an HP-5MS capillary column (stationary phase: 5%–phenyl-methylpolysiloxane; length: 30 m; ID: 0.25 mm; film thickness: 0.25 µm), coupled to an Agilent 5973 Inert MS detector operated in the full scan mode (*m/z* 40–550) at a frequency of 3.9 Hz and with the EI ion source and quadrupole mass filter temperatures kept, respectively, at 200 °C and 250 °C. Helium was used as carrier gas at a flow rate of 1 mL/min. The injector temperature was 250 °C, and the temperature ramp raised the column temperature from 70 °C to 280 °C: 70 °C for 1 min; 10 °C/min until reaching 170 °C; and 30 °C for 1 min until reaching 280 °C. Then, it was held at 280 °C for 5 min. The solvent delay was 4 min. Metabolites were identified by comparing their EI mass spectra at 70 eV with mass spectra collected in the NIST 20 mass spectral library (available online: https://www.nist.gov/srd/nist-standard-reference-database-1a, accessed on 25 June 2025). Moreover, the identification was supported by the Kovats retention index (RI) calculated for each metabolite by the Kovats equation, using the standard *n*-alkane mixture in the range C7–C40.

### 4.7. Antifungal Assay

The antifungal activity of *G. ulicina* extracts (*n*-hexane and DCM) was evaluated against three phytopathogenic fungi: *F. oxysporum*, *A. alternata*, and *B. cinerea*. These fungi are responsible for significant agricultural losses affecting crops worldwide. Dried organic extracts were initially dissolved in dimethyl sulfoxide (DMSO) to prepare stock solutions, diluted with sterile distilled water to achieve the desired working concentrations (1 mg/mL), ensuring that the final DMSO concentration in the assay was ≤1% (*v/v*).

A modified agar plate diffusion method was used to assess antifungal activity. Fungal spores were cultured on potato dextrose agar (PDA) plates. After seven days of incubation at 28 °C, 1 mL of each extract solution was aseptically added to sterile PDA medium and spread evenly. A fungal inoculum was then placed at the center of the treated medium. Control plates containing only 1% DMSO (without extract) were also prepared. Each treatment was conducted in triplicate. All Petri dishes were incubated at 28 °C for 8 days. Antifungal activity was determined by measuring the colony diameter, and results were expressed as mean colony diameter (cm). The percentage of growth inhibition (% I) was calculated according to the following formula:

$$\%\;Inhibition=\frac{D_{control}-D_{treatment}}{D_{control}}\times 100$$

### 4.8. Leaf Puncture Assay

The phytotoxic activity of *G. ulicina* organic extracts (*n*-hexane and dichloromethane, DCM) was evaluated using a detached leaf puncture assay. Fully expanded leaves of *Euphorbia peplus* L. and *Oxalis corniculata* L. were collected from plants growing naturally in the field. These species were selected due to their prevalence as weeds and their reported sensitivity to phytotoxic compounds.

Detached leaves were placed in sterile Petri dishes (90 mm diameter) lined with moistened filter paper to maintain humidity. Small punctures were made in the central area of each leaf using a sterile needle to facilitate absorption. The extracts were dissolved in 4% methanol (MeOH) and tested at concentrations of 0.5, 1.0, and 2.0 mg/mL [41]. A 20 μL aliquot of each solution was applied directly onto the punctured area, while 4% MeOH served as the negative control [42].

All treatments were performed in triplicate, and leaves were incubated under laboratory conditions at 25 ± 2 °C. The development of necrotic lesions was monitored and documented 24–28 h post-treatment. Necrosis was evaluated based on lesion diameter and intensity and scored using a semi-quantitative scale (0–3), with higher values indicating greater damage.

### 4.9. Statistical Analysis

All experiments were carried out in triplicate, and data are presented as means ± standard deviation (SD). The effects of plant part and extract concentration on the necrotic area (mm^2^) induced by *n*-hexane and DCM extracts were evaluated using two-way analysis of variance (ANOVA), followed by Tukey’s HSD test for multiple comparisons. Statistical analyses were performed using XLSTAT software (Addinsoft SARL, New York, NY, USA). Differences were considered statistically significant at *p* ≤ 0.05.

### 4.10. Molecular Docking Analysis

A structure-based molecular docking approach was employed to evaluate the herbicidal potential of 38 phytochemical constituents isolated from *G. ulicina* against three key plant enzymes involved in essential metabolic pathways: acetohydroxyacid synthase (AHAS; PDB ID: 1YHZ), 4-hydroxyphenylpyruvate dioxygenase (HPPD; PDB ID: 6J63), and protoporphyrinogen oxidase (PPO; PDB ID: 1SEZ). These enzymes are well-established targets of commercial herbicides and were selected based on their relevance in branched-chain amino acid biosynthesis, plastoquinone production, and chlorophyll biosynthesis, respectively.

#### 4.10.1. Ligand Preparation

The 38 phytochemical compounds were retrieved in SDF format from the PubChem database (https://pubchem.ncbi.nlm.nih.gov, accessed on 17 July 2025) using their respective Compound IDs (CIDs). The molecular structures were energy-minimized and converted to the PDBQT format required for docking using Open Babel. Partial charges were assigned, and torsional bonds were defined using AutoDockTools. For each compound, the geometry was optimized using the MMFF94 force field to ensure a biologically relevant conformation.

#### 4.10.2. Protein Preparation

The crystal structures of the three target enzymes were downloaded from the Protein Data Bank (https://www.rcsb.org, accessed on 17 July 2025). Water molecules, irrelevant ions, and co-crystallized ligands were removed unless the ligand was essential for defining the active site or functionally necessary. Hydrogen atoms were added, and Gasteiger charges were computed using AutoDockTools. The prepared protein structures were saved in PDBQT format for subsequent docking procedures.

#### 4.10.3. Validation by Re-Docking

Prior to docking the phytochemicals, a re-docking procedure was conducted to validate the reliability of the docking protocol. The native co-crystallized ligand of each enzyme was extracted and re-docked into its respective active site using AutoDock Vina (version 1.1.2). The resulting ligand poses were compared to the original crystallographic orientations, and root-mean-square deviation (RMSD) values were calculated. RMSD values below 1.5 Å were considered acceptable, confirming the validity of the docking settings.

#### 4.10.4. Molecular Docking

Docking simulations were carried out using AutoDock Vina (version 1.1.2) with default parameters. A grid box was defined to encompass the active site of each enzyme, centered on the coordinates of the co-crystallized ligand. Each phytochemical compound was docked individually to all three enzyme targets. For each docking run, the top-scoring binding poses based on the Vina binding affinity (expressed in kcal/mol) were selected for further analysis.

#### 4.10.5. Visualization and Interaction Analysis

Docking results were analyzed and visualized using Discovery Studio Visualizer (version 3.0) to examine the binding interactions between the top-ranked phytochemicals and the active site residues of the enzymes. Key interactions such as hydrogen bonding, and hydrophobic contacts were identified to interpret the binding mode and potential inhibitory effects of each compound.

## Figures and Tables

**Table 1:** Chromatographic peak area percentage of compounds identified via GC-MS in crude extracts (*n*-hexane and dichloromethane) of aerial part and roots of *G. ulicina*. (RI: Kovats retention index; TMS: trimethylsilyl function).

Compound	RI	Aerial Part		Roots	
		*n*-Hexane Extract	Dichloromethane Extract	*n*-Hexane Extract	Dichloromethane Extract
Benzyl alcohol, TMS	1168		0.91		
Phenylethyl alcohol, TMS	1234	0.41	2.18		
Octanoic acid, TMS	1265	0.31		0.11	
Glycerol, 3TMS	1283		0.68		0.55
Carvacrol, TMS	1338			0.11	
Nonanoic acid, TMS	1362			0.16	
Eugenol, TMS	1479	0.58		0.22	
Hydroxy-5-methoxy-3-methyl-5-oxopentanoic acid, 2TMS	1508		5.17		
Methyl laurate	1526	1.41			
Vanillin, TMS	1549				1.07
2,4-Di-tert-butylphenoxytrimethylsilane	1555			1.07	
Elemicin	1569			2.94	
3-Hydroxy-3-methylglutaric acid, 3TMS	1613				3.07
Vanillyl alcohol, 2TMS	1648				0.43
Lauric acid, TMS	1657	8.10		0.32	
8-Carbomethoxyoctanoic acid, TMS	1680	2.10	6.86		
2,6-Dimethoxyhydroquinone, 2TMS	1689				3.03
Syringaldehyde, TMS	1720		2.99		46.77
Methyl 3,4-dihydroxybenzoate, 2TMS	1734		1.95		
Vanillic acid, 2TMS	1775		3.99		2.02
Azelaic acid, 2TMS	1800		29.96		6.11
Myristic acid, TMS	1849	8.27			
Coniferyl aldehyde, TMS	1863				0.87
Syringic acid, 2TMS	1910				9.32
Isoprunetin, 2TMS	1926		9.79		
Methyl palmitate	1931	30.34		2.34	
Ferulic acid, methyl ester, TMS	1969	6.08	5.22		
Methyl caffeate, 2TMS	2028		30.29		
Sinapaldehyde, TMS	2036				17.66
Sinapyl alcohol, 2TMS	2095				3.16
Palmitic acid, TMS	2051	39.25		17.39	
Linolelaidic acid, methyl ester	2108			15.24	
Oleic acid, TMS	2239			59.53	
Stearic acid, TMS	2242	3.15			
1-Monolinolein, 2TMS	2769			0.55	
Daidzein, 2TMS	2976				3.49
Genistein, 3TMS	3000				2.03

**Table 2:** Antifungal activity (% inhibition) of *Genista ulicina* extracts (*n*-hexane, DCM; 1 mg mL^−1^) against phytopathogenic fungi.

Plant Part			% Inhibition	
		*F. oxysporum*	*A. alternata*	*B. cinerea*
Aerial	*n*-Hexane	100 ± 0.0 ^a^	87 ± 1.6 ^b^	100 ± 0.0 ^a^
	DCM	100 ± 0.0 ^a^	67 ± 2.1 ^c^	100 ± 0.0 ^a^
Root	*n*-Hexane	96.4 ± 1.5 ^b^	98.2 ± 1.0 ^a^	100 ± 0.0 ^a^
	DCM	94.7 ± 1.8 ^b^	97.1 ± 1.2 ^a^	100 ± 0.0 ^a^

Values are expressed as mean ± SD. Different letters indicate statistically significant differences at *p* ≤ 0.05 based on Tukey’s HSD test. The same letters indicate no significant difference.

**Table 3:** Necrotic area (mm^2^) induced by *G. ulicina n*-hexane and dichloromethane (DCM) extracts of aerial and root parts on *O. corniculata* leaves at different concentrations.

Plant Part		Necrotic Area (mm^2^)			
		0.5 mg/mL	1 mg/mL	2 mg/mL	Control
Aerial	*n*-Hexane	12.33 ± 0.62 ^bcd^	25.33 ± 0.70 ^bc^	48.50 ± 0.72 ^a^	0.50 ± 0.10 ^e^
	DCM	10.90 ± 0.20 ^cd^	22.20 ± 0.36 ^cd^	42.60 ± 0.57 ^ab^	0.47 ± 0.06 ^e^
Root	*n*-Hexane	14.27 ± 0.35 ^bc^	30.40 ± 0.62 ^b^	55.30 ± 0.75 ^a^	0.50 ± 0.10 ^e^
	DCM	13.10 ± 0.30 ^bc^	27.73 ± 0.40 ^b^	51.30 ± 0.40 ^a^	0.47 ± 0.06 ^e^

Values are expressed as mean ± SD. Different letters indicate statistically significant differences at *p* ≤ 0.05 based on Tukey’s HSD test. Same letters mean no significant difference.

**Table 4:** Necrotic area (mm^2^) induced by *G. ulicina n*-hexane and dichloromethane (DCM) extracts of aerial and root parts on *Euphorbia peplus* leaves at different concentrations.

Plant Part		Necrotic Area (mm^2^)			
		0.5 mg/mL	1 mg/mL	2 mg/mL	Control
Aerial	*n*-Hexane	5.90 ± 0.20 ^cd^	12.43 ± 0.25 ^bc^	24.33 ± 0.56 ^ab^	0.20 ± 0.00 ^e^
	DCM	4.10 ± 0.55 ^d^	6.40 ± 0.26 ^cd^	10.77 ± 0.26 ^c^	0.27 ± 0.06 ^e^
Root	*n*-Hexane	7.13 ± 0.25 ^c^	14.97 ± 0.81 ^b^	26.57 ± 0.57 ^a^	0.27 ± 0.06 ^e^
	DCM	6.37 ± 0.30 ^cd^	13.57 ± 0.15 ^bc^	25.40 ± 0.41 ^a^	0.20 ± 0.00 ^e^

Values are expressed as mean ± SD. Different letters indicate statistically significant differences at *p* ≤ 0.05 based on Tukey’s HSD test. Same letters mean no significant difference.

**Table 5:** Docking scores (binding energies in kcal/mol) and interaction profiles of top-ranked phytochemicals from *Genista ulicina* extracts against selected herbicide target enzymes (AHAS, HPPD, and PPO) as predicted by molecular docking.

			Binding Energy (Kcal/mol)	Hydrogen Interactions (Distance Å)	Hydrophobic Interactions
**AHAS (1YHZ)**	Co-crystallized ligand	1CS	−8.5	Gly508 (2.56), Gly508 (2.84), Asp375 (2.54), Arg373 (2.52), Gly509 (3.23), Arg377 (2.60)	Trp574, Met570
	Best docked compounds	Linolelaidic acid, methyl ester	−7.6	Gly509 (2.65), Arg373 (2.23)	Leu332 (2) Val378, Arg377 (2) Met490 (2), Met351, His352, Met570 (2)
		1-Monolinolein	−6.8	Gly509 (2.41), Arg377 (2.78), Asp375 (2.95), Asp375 (2.26), Asp376 (3.08)	Val571 (2), Tyr579, Met570, Trp574 (3), Arg377
		Palmitic acid	−6.7	Ser540 (2.51), Gly569 (2.67), Met570 (2.49)	Met513, Val485, Met490 (2), Arg377 (2), Met351 (2) His352
**HPPD** **(6J63)**	Co-crystallized ligand	NDT	−6.9	His226 (2.63), His308 (3.26), His308 (3.35), Lys421 (2.92)	Phe424 (2)
	Best docked compounds	1-Monolinolein	−7.7	His226 (2.59), His308 (2.94), Glu252 (2.72), Ser267 (3.26)	Phe424, Phe381 (3), Met335, Leu265, Phe392
		Linolelaidic acid, methyl ester	−7.5	Glu252 (2.95), Ser267 (3.22)	Pro338, Leu368 (2), Met335 (2), Leu427, Phe381 (2), Phe424 (2), His308, Lys421, Val269, Phe419
		Stearic acid	−7.3	Gln293 (2.63)	Pro280, Val228, His308, Phe381, Phe424 (3)
**PPO** **(1SEZ)**	Co-crystallized ligand	OMN	−8.1	Arg98 (2.33), Gly178 (2.65)	Leu372, Leu356, Phe392 (2), Leu334
	Best docked compounds	1-Monolinolein	−8.7	Gly354 (2.92), Arg98 (2.64)	Trp435, Ala438, Leu334, Ala66 (3), Phe439, Lys51, Val475, Phe392
		Linolelaidic acid, methyl ester	−8.4	Gly354 (2.91), Gly354 (3.06)	Ala478, Lys51, Ala66 (2), Phe439 (3), Phe392 (2), Arg98, Leu356
		Palmitic acid, methyl ester	−8.1	Gly354 (3.13), Gly354 (3.24)	Arg98, Leu356 (2), Leu372, he392 (4), Phe439, Ala438, Lys51, Trp435

## Data Availability

The original contributions presented in this study are included in the article/Supplementary Materials. Further inquiries can be directed at the corresponding author.

## Supplementary Materials

The following supporting information can be downloaded at: https://www.mdpi.com/article/10.3390/toxins17090452/s1. Figure S1. TLC of *n*-hexane and dichloromethane extracts of *G. ulicina* roots (spots A and B, respectively) and aerial part (spots C and D, respectively) eluted with chloroform-isopropanol 9:1. The spots were visualized by exposure to UV radiation (254 nm). Figure S2. ^1^H NMR spectrum of *G. ulicina* aerial part *n*-hexane extract recorded at 400 MHz in CDCl_3_. Figure S3. ^1^H NMR spectrum of *G. ulicina* aerial part dichloromethane extract recorded at 400 MHz in CD_3_OD. Figure S4. ^1^H NMR spectrum of *G. ulicina* roots *n*-hexane extract recorded at 400 MHz in CDCl_3_. Figure S5. ^1^H NMR spectrum of *G. ulicina* roots dichloromethane extract recorded at 400 MHz in CD_3_OD. Figure S6. Annotated total ion chromatograms (TICs) of crude extracts (*n*-hexene and dichloromethane), after derivatization with *N,O*-bis(trimethylsilyl)trifluoroacetamide (BSTFA), of aerial parts of *Genista ulicina*. Figure S7. Annotated total ion chromatograms (TICs) of crude extracts (*n*-hexene and dichloromethane), after derivatization with *N,O*-bis(trimethylsilyl)trifluoroacetamide (BSTFA), of roots of *Genista ulicina*.

## Abbreviations

The following abbreviations are used in this manuscript:

DCM	Dichloromethane

## References

[1] Rani K., Dhania G. (2014). Bioremediation and biodegradation of pesticide from contaminated soil and water—A noval approach. Int. J. Curr. Microbiol. App. Sci..

[2] Rajmohan K.-S., Chandrasekaran R., Varjani S. (2020). A review on occurrence of pesticides in environment and current technologies for their remediation and management. Indian J. Microbiol..

[3] Nega A. (2014). Review on concepts in biological control of plant pathogens. J. Biol. Agric. Healthc..

[4] Zatout R., Benarbia R., Yalla I. (2024). Phytochemical profiles and antimicrobial activities of *Phellinus* mushroom: Implications for agricultural health and crop protection. Agric. Res. J..

[5] Hlaili H., Zorrilla J.-G., Salvatore M.-M., Abassi M., Russo M.-T., Martínez-González M.-I., Masi M. (2025). Metabolite Screening From *Pinus pinea* Needles reveals (+)-isocupressic acid as a key phytotoxin for weed management. Phytochem. Anal..

[6] Lengai G.-M., Muthomi J.-W., Mbega E.-R. (2020). Phytochemical activity and role of botanical pesticides in pest management for sustainable agricultural crop production. Sci. Afr..

[7] Khursheed A., Rather M.-A., Jain V., Rasool S., Nazir R., Malik N.-A., Majid S.-A. (2022). Plant based natural products as potential ecofriendly and safer biopesticides: A comprehensive overview of their advantages over conventional pesticides, limitations and regulatory aspects. Microb. Pathog..

[8] Vlaiculescu A., Varrone C. (2022). Sustainable and eco-friendly alternatives to reduce the use of pesticides. Pesticides in the Natural Environment.

[9] Verma N.-S., Kuldeep D.-K., Chouhan M., Prajapati R., Singh S.-K. (2023). A review on eco-friendly pesticides and their rising importance in sustainable plant protection practices. Int. J. Plant Soil Sci..

[10] He B., Hu Y., Wang W., Yan W., Ye Y. (2022). The progress towards novel herbicide modes of action and targeted herbicide development. Agronomy.

[11] Poslinski H., Hatley M., Tramell J., Song B.-H. (2025). Harnessing phytochemicals in sustainable and green agriculture. J. Agric. Food Res..

[12] Del Prete S., Pagano M. (2024). Enzyme inhibitors as multifaceted tools in medicine and agriculture. Molecules.

[13] Aourahoun K.-A.-K., Fazouane F., Benayache S., Bettache Z.-H., Benayad T., Denni N. (2019). Antioxidant and anti-inflammatory activity of phenolic extracts of *Genista ferox* (Fabaceae). Pak. J. Pharm. Sci..

[14] Laranjeira I.-M., Dias A.-C.-P., Pinto-Ribeiro F.-L. (2023). *Genista tridentata* phytochemical characterization and biological activities: A systematic review. Biology.

[15] Gibbs P.-E. (1974). Taxonomic notes on some Canry Island in North African species of *Cytisus* and *Genista*. Lagascalia.

[16] Bacchetta G., Brullo S., Velari T.-C., Chiapella L.-F., Kosovel V. (2011). Taxonomic notes on the *Genista ephedroides* group (Fabaceae) from the Mediterranean area. Novon J. Bot. Nomencl..

[17] Zheng Z., Scott S., Lukas W., Webb M. (2000). A greedy algorithm for aligning DNA sequences. J. Comput. Biol..

[18] Dananjaya S.-H.-S., Udayangani R.-M.-C., Shin S.-Y., Edussuriya M., Nikapitiya C., Lee J., De Zoysa M. (2017). In vitro and in vivo antifungal efficacy of plant based lawsone against *Fusarium oxysporum* species complex. Microbiol. Res..

[19] Rongai D., Pulcini P., Pesce B., Milano F. (2015). Antifungal activity of some botanical extracts on *Fusarium oxysporum*. Open Life Sci..

[20] Xu J., Zhao X., Han X., Du Y. (2007). Antifungal activity of oligochitosan against *Phytophthora capsici* and other plant pathogenic fungi in vitro. Pestic. Biochem. Physiol..

[21] Tang C.-S., Young C.-C. (1982). Collection and identification of allelopathic compounds from the undisturbed root system of *Bigalta limpograss* (*Hemarthria altissima*). Plant Physiol..

[22] Hao Z.-P., Wang Q., Christie P., Li X.L. (2007). Allelopathic potential of watermelon tissues and root exudates. Sci. Hortic..

[23] Araniti F., Lupini A., Sorgonà A., Conforti F., Marrelli M., Statti G.-A., Abenavoli M.-R. (2013). Allelopathic potential of *Artemisia arborescens*: Isolation, identification and quantification of phytotoxic compounds through fractionation-guided bioassays. Nat. Prod. Res..

[24] Khan A.-S., Sakina, Nasrullah A., Ullah S., Ullah Z., Khan Z., Di I.-U. (2023). An overview on phytotoxic perspective of ionic liquids and deep eutectic solvents: The role of chemical structure in the phytotoxicity. Chem. Biomol. Eng. Rev..

[25] Dezfulian M.-H., Foreman C., Jalili E., Pal M., Dhaliwal R.-K., Roberto D.-K.-A., Imre K.-M., Kohalmi S.-E., Crosby W.-L. (2017). Acetolactate synthase regulatory subunits play divergent and overlapping roles in branched-chain amino acid synthesis and Arabidopsis development. BMC Plant Biol..

[26] Thomson M.-K., Krämer W., Schirmer U. (2007). Acetohydroxyacid synthase inhibitors (AHAS/ALS). Modern Crop Protection Compounds.

[27] Kraehmer H., van Almsick A., Beffa R., Dietrich H., Eckes P., Hacker E., Hain R., Strek H.J., Stuebler H., Willms L. (2014). Herbicides as Weed Control Agents: State of the Art: II. Recent Achievements. Plant Physiol..

[28] Liu S., Qin S., Zhang T., Zhang H., Zhu J., Li X., Li Y., Zhao F. (2025). Basing target enzyme study the enantioselective bioactivity action mechanism of flusulfinam, a novel HPPD inhibitor herbicide. Pestic. Biochem. Physiol..

[29] Koch M., Breithaupt C., Kiefersauer R., Freigang J., Huber R., Messerschmidt A. (2004). Crystal structure of protoporphyrinogen IX oxidase: A key enzyme in haem and chlorophyll biosynthesis. EMBO J..

[30] Fan Q., Shen Y., Yang Y., Zhang Q. (2024). A review of remediation strategies for diphenyl ether herbicide contamination. Toxics.

[31] Takács E., Lázár D., Siakwa A., Klátyik S., Mörtl M., Kocsányi L., Barócsi A., Lenk S., Lengyel E., Székács A. (2024). Ecotoxicological evaluation of safener and antimicrobial additives in isoxaflutole-based herbicide formulations. Toxics.

[32] Geoffroy L., Teisseire H., Couderchet M., Vernet G. (2002). Effect of oxyfluorfen and diuron alone and in mixture on antioxidative enzymes of *Scenedesmus obliquus*. Pestic. Biochem. Physiol..

[33] Halfaoui F., Touahria S. (2018). Consequences of the degradation of plant formations in the region of Tamezguida, wilaya of Medea. Agrobiologia.

[34] Mangold J., Parkinson H. (2013). Plant identification basics. Mont. State Univ. Ext. Mt. Guide.

[35] Murray M.-G., Thompson W.-F. (1980). Rapid isolation of high molecular weight plant DNA. Nucleic Acids Res..

[36] Gardes M., Bruns T.-D. (1993). ITS primers withenhanced specificity for basidiomycetesapplication to the identification ofmycorrhizae and rusts. Mol. Ecol..

[37] White T.J., Bruns T., Lee S., Taylor J., Innis M.A., Gelfand D.H., Sninsky J.J., White T.J. (1990). Amplification and direct sequencing of fungal ribosomal RNA genes for phylogenetics. PCR Protocols: A Guide to Methods and Applications.

[38] Zatout R., Christopher B., Cherfia R., Chaoua S., Chaouche N.-K. (2023). A new record of *Agaricus litoralis*, a rare edible macro-fungus from Eastern Algeria. Mycopath.

[39] Zatout R., Kacem Chaouche N. (2023). Antibacterial activity screening of an edible mushroom *Agaricus litoralis*. Int. J. Bot. Stud..

[40] Salvatore M.M., Pappalardo C., Suarez E.G.P., Salvatore F., Andolfi A., Gesuele R., Galdiero E., Libralato G., Guida M., Siciliano A. (2024). Ecotoxicological and metabolomic investigation of chronic exposure of *Daphnia magna* (Straus, 1820) to yttrium environmental concentrations. Aquat. Toxicol..

[41] Zatout R., Cimmino A., Cherfia R., Chaouche N.-K. (2021). Isolation of tyrosol the main phytotoxic metabolite produced by the edible fungus *Agaricus litoralis*. Egypt. J. Chem..

[42] Masi M., Sautua F., Zatout R., Castaldi S., Arrico L., Isticato R., Evidente A. (2021). Phaseocyclopentenones A and B, phytotoxic penta-and tetrasubstituted cyclopentenones produced by *Macrophomina phaseolina*, the causal agent of charcoal rot of soybean in Argentina. J. Nat. Prod..

