# Supplementary material for: Exploring the Potential of Genista ulicina Phytochemicals as Natural Biocontrol Agents: A Comparative In Vitro and In Silico Analysis
Source: Toxins (Basel). 2025 Sep 6;17(9):452. doi: 10.3390/toxins17090452 (PMC12474477; doi:10.3390/toxins17090452)
Supplement: Supplementary file 1 [file toxins-17-00452-s001.zip › toxins-3796692-supplementary.pdf]

Supplementary Materials

Exploring the Potential of *Genista ulicina* Phytochemicals as Natural Biocontrol Agents: A Comparative In vitro and In Silico Analysis

Roukia Zatout [1,*], Ouided Benslama [2], Fatima Zohra Makhlouf [3], Alessio Cimmino [4], Maria Michela Salvatore [5], Anna Andolfi [4], Radhia Manel Kolla [1] and Marco Masi [4,*]

[1] Department of Microbial Biotechnology, Faculty of Natural and Life Science, University of Blida 1, Ouled Yaich 09000, Blida, Algeria; radhia.manel.kolla@umc.edu.dz
[2] Department of Natural and Life Sciences, Faculty of Exact Sciences and Natural and Life Sciences, Larbi Ben M'Hidi University, Oum El Bouaghi 04000, Algeria; benslama.wided@hotmail.fr
[3] Higher National School of Biotechnology Taoufik Khaznadar, Nouveau Pôle Universitaire Ali Mendjeli, BP. E66, Constantine 25100, Algeria; makhlouf_f.zohra@umc.edu.dz
[4] Department of Chemical Sciences, University of Naples Federico II, 80126 Naples, Italy; alessio.cimmino@unina.it (A.C.); andolfi@unina.it (A.A.)
[5] Department of Veterinary Medicine and Animal Production, University of Naples Federico II, 80137 Naples, Italy; mariamichela.salvatore@unina.it
* Correspondence: roukia.zatout@umc.edu.dz (R.Z.); marco.masi@unina.it (M.M.)

Contents

Figure S1. TLC of *n*-hexane and dichloromethane extracts of *G. ulicina* roots (spots A and B, respectively) and aerial part (spots C and D, respectively) eluted with chloroform-isopropanol 9:1. The spots were visualized by exposure to UV radiation (254 nm).

Figure S2. ^{1}H NMR spectrum of *G. ulicina* aerial part *n*-hexane extract recorded at 400 MHz in $CDCl_3$.

Figure S3. ^{1}H NMR spectrum of *G. ulicina* aerial part dichloromethane extract recorded at 400 MHz in CD_3OD.

Figure S4. ^{1}H NMR spectrum of *G. ulicina* roots *n*-hexane extract recorded at 400 MHz in $CDCl_3$.

Figure S5. ^{1}H NMR spectrum of *G. ulicina* roots dichloromethane extract recorded at 400 MHz in CD_3OD.

Figure S6. Annotated Total Ion Chromatograms (TICs) of crude extracts (*n*-hexene and dichloromethane), after derivatization with *N,O*-bis(trimethylsilyl)trifluoroacetamide (BSTFA), of aerial parts of *Genista ulicina*.

Figure S7. Annotated Total Ion Chromatograms (TICs) of crude extracts (*n*-hexene and dichloromethane), after derivatization with *N,O*-bis(trimethylsilyl)trifluoroacetamide (BSTFA), of roots of *Genista ulicina*.

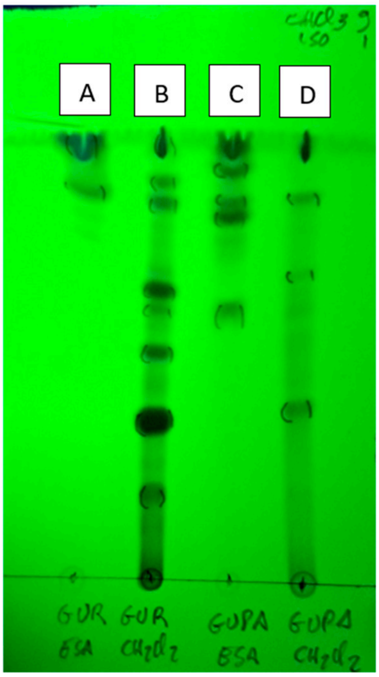

Figure S1. TLC of *n*-hexane and dichloromethane extracts of *G. ulicina* roots (spots A and B, respectively) and aerial part (spots C and D, respectively) eluted with chloroform-isopropanol 9:1. The spots were visualized by exposure to UV radiation (254 nm).

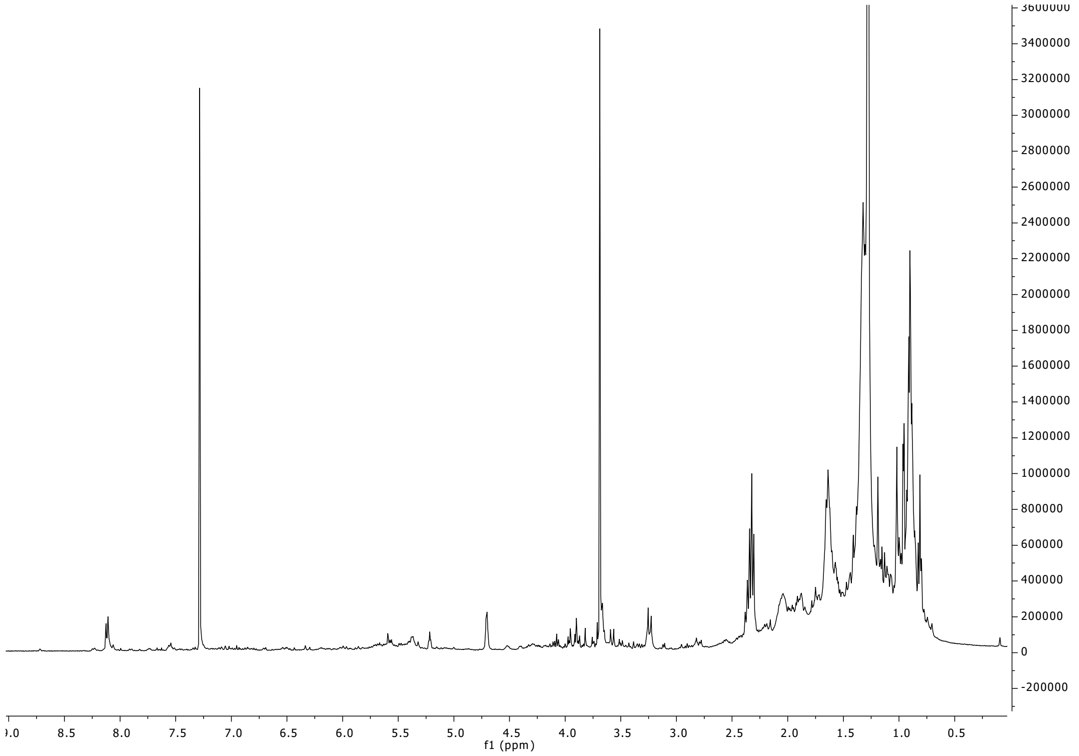

Figure S2. ^{1}H NMR spectrum of *G. ulicina* aerial part *n*-hexane extract recorded at 400 MHz in $CDCl_3$.

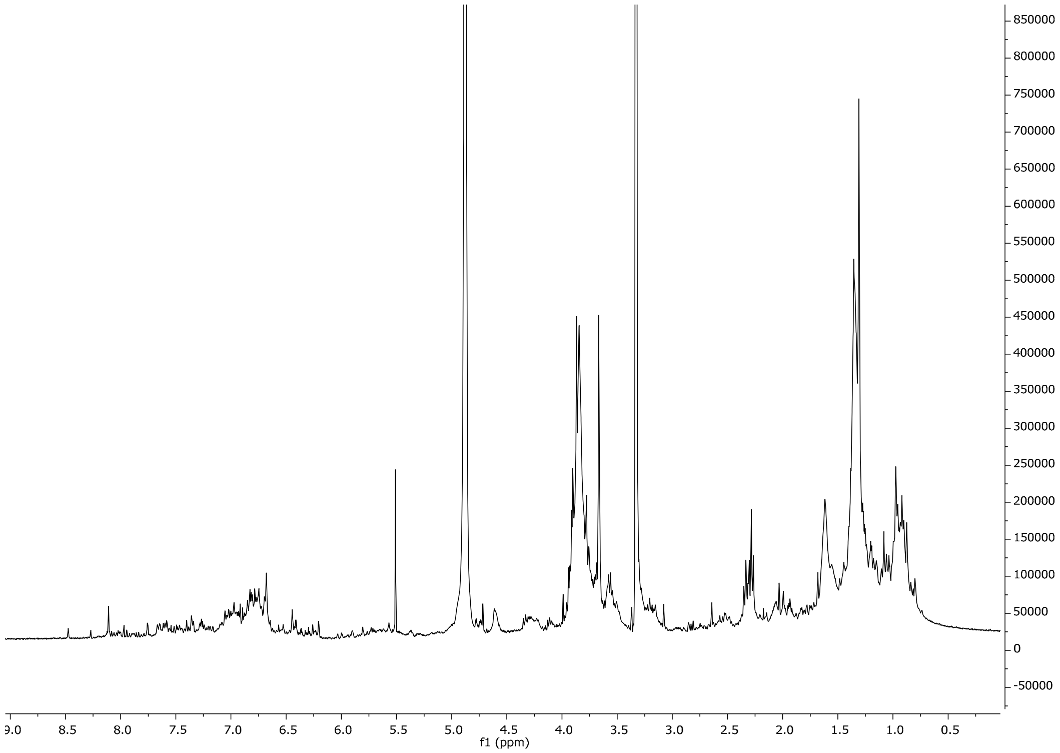

Figure S3. ^{1}H NMR spectrum of *G. ulicina* aerial part dichloromethane extract recorded at 400 MHz in CD_3OD.

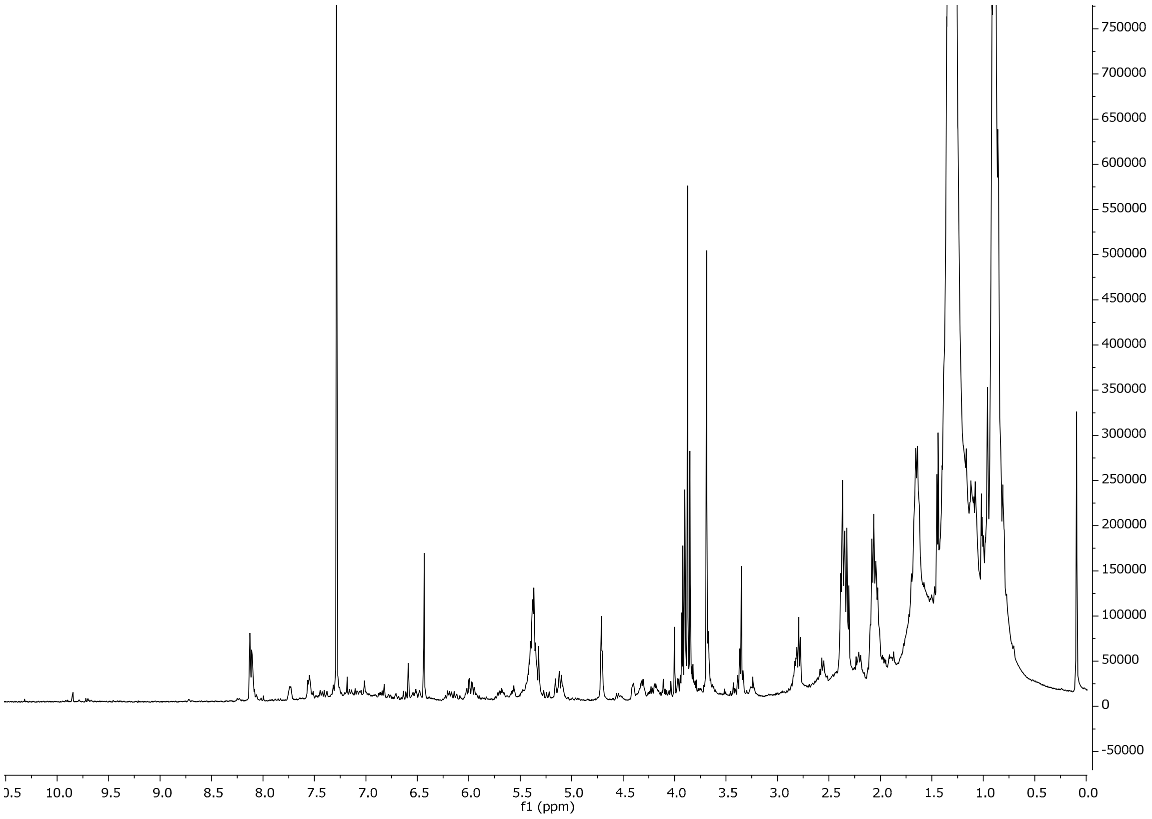

Figure S4. ^{1}H NMR spectrum of *G. ulicina* roots *n*-hexane extract recorded at 400 MHz in $CDCl_3$.

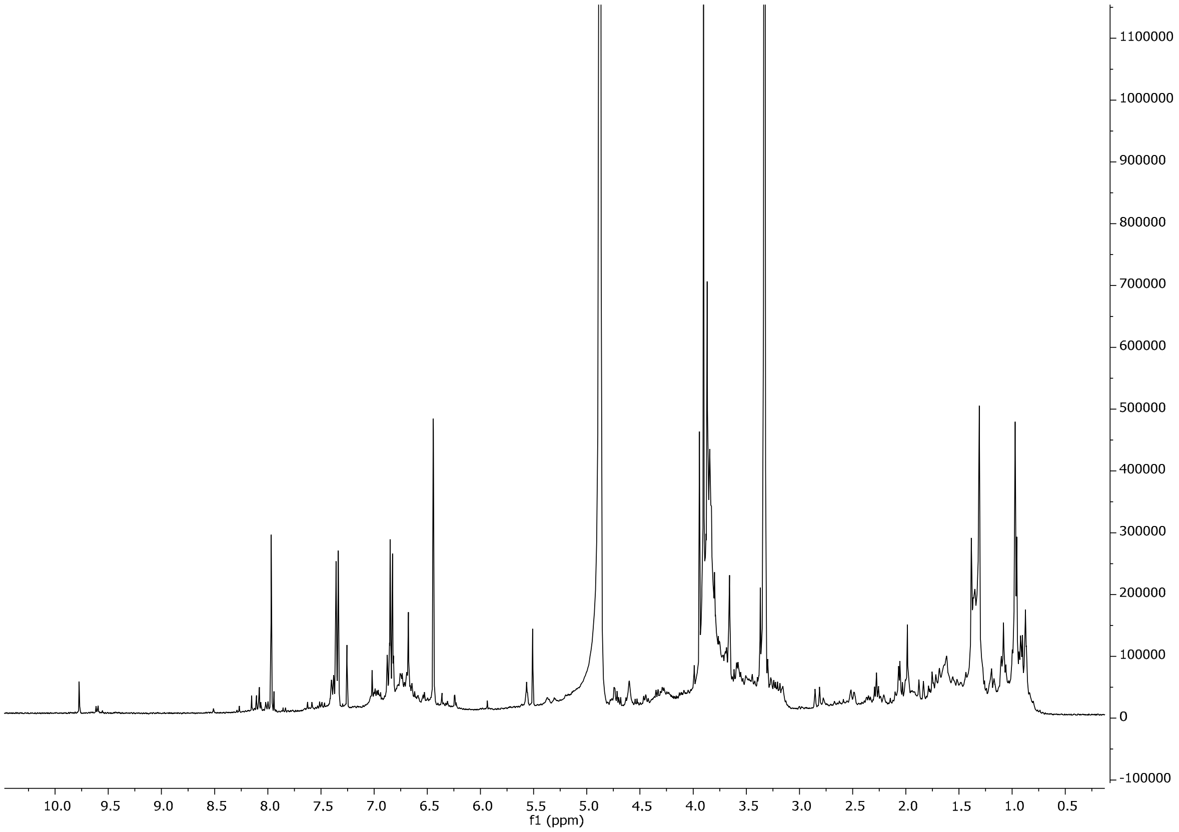

Figure S5. ^{1}H NMR spectrum of *G. ulicina* roots dichloromethane extract recorded at 400 MHz in CD_3OD.

n-Hexane extract

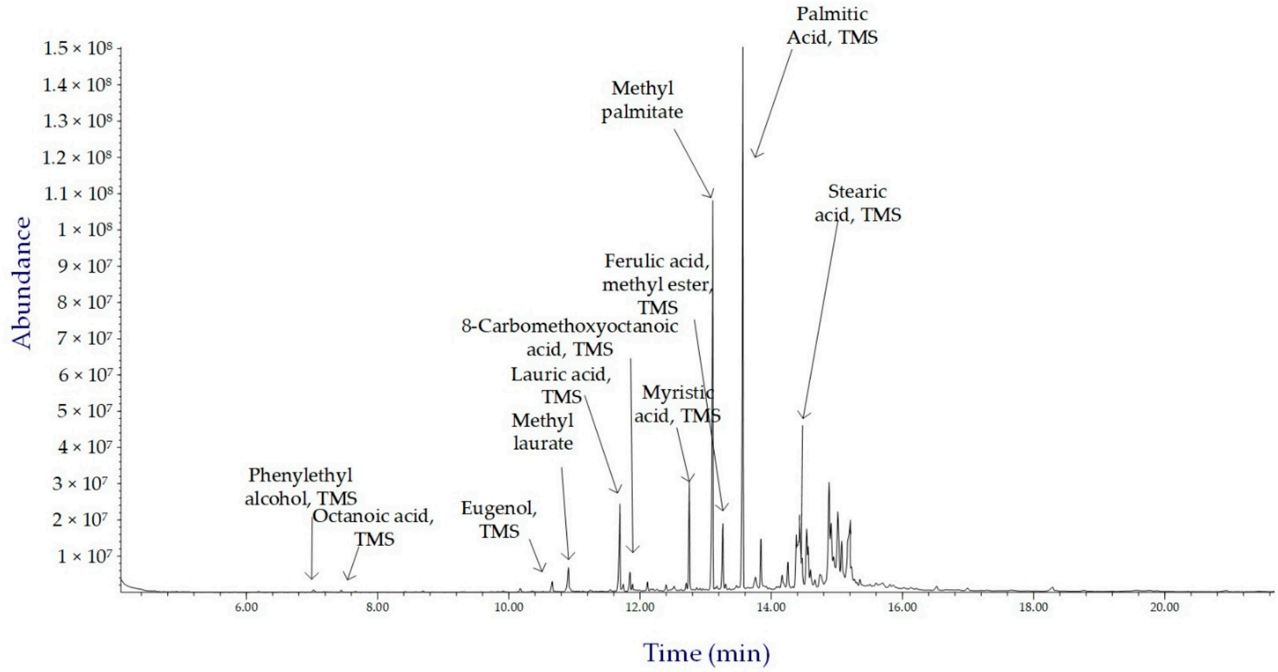

Dichloromethane extract

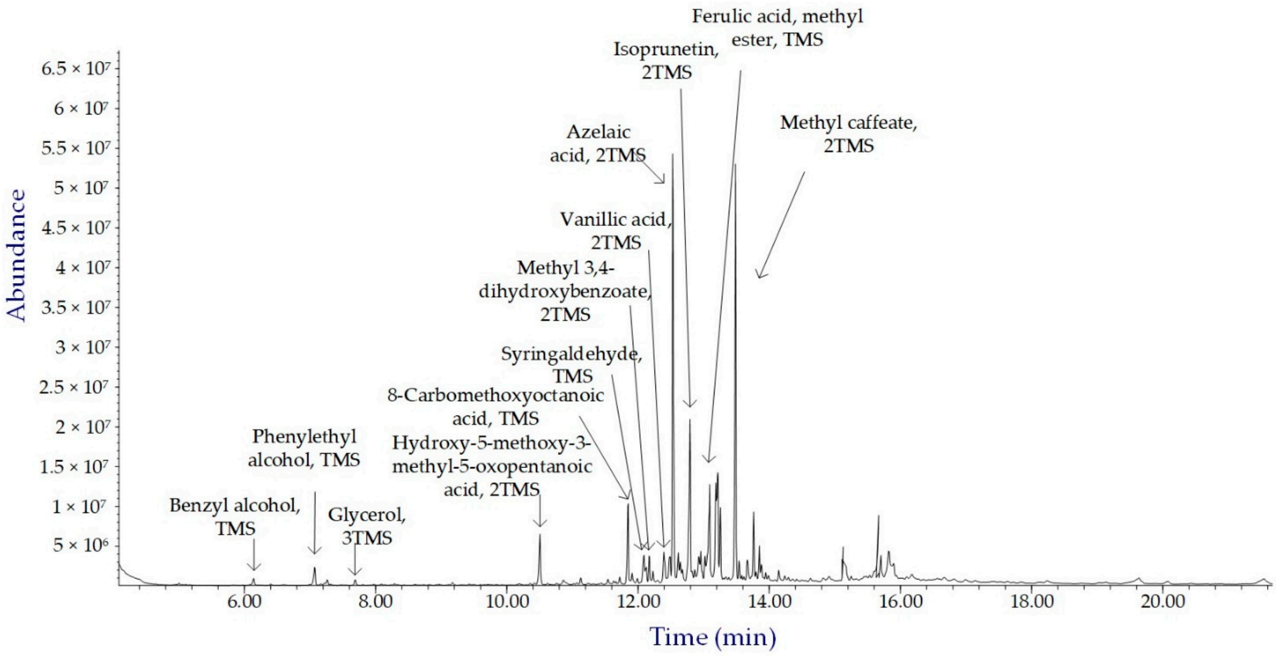

Figure S6. Annotated Total Ion Chromatograms (TICs) of crude extracts (*n*-hexane and dichloromethane), after derivatization with *N*,*O*-bis(trimethylsilyl)trifluoroacetamide (BSTFA), of aerial parts of *Genista ulicina*.

n-Hexane extract

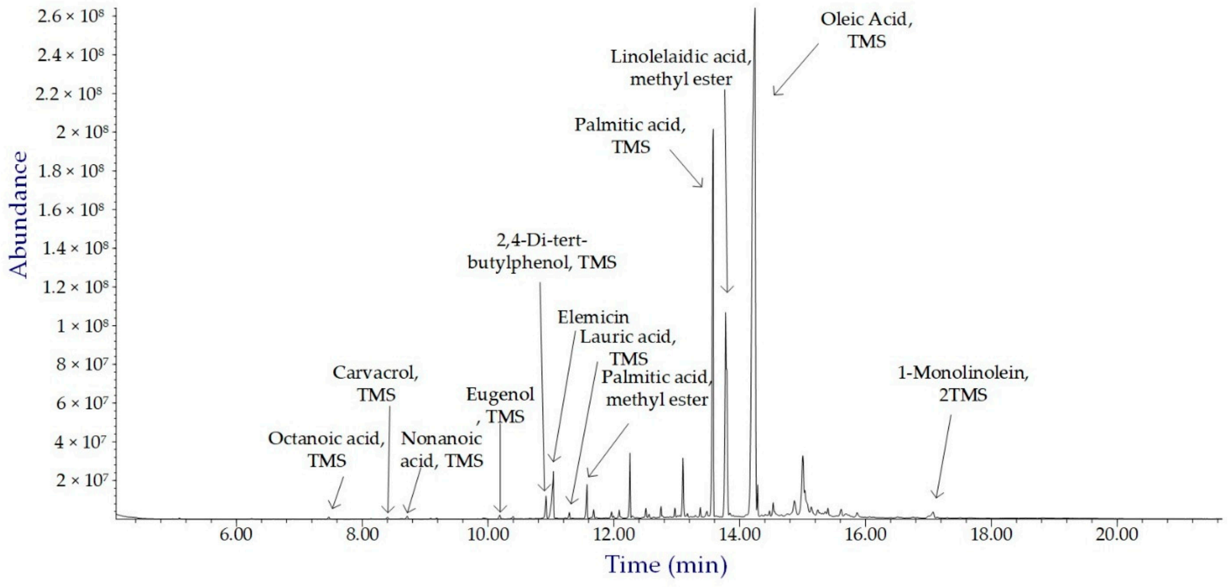

Dichloromethane extract

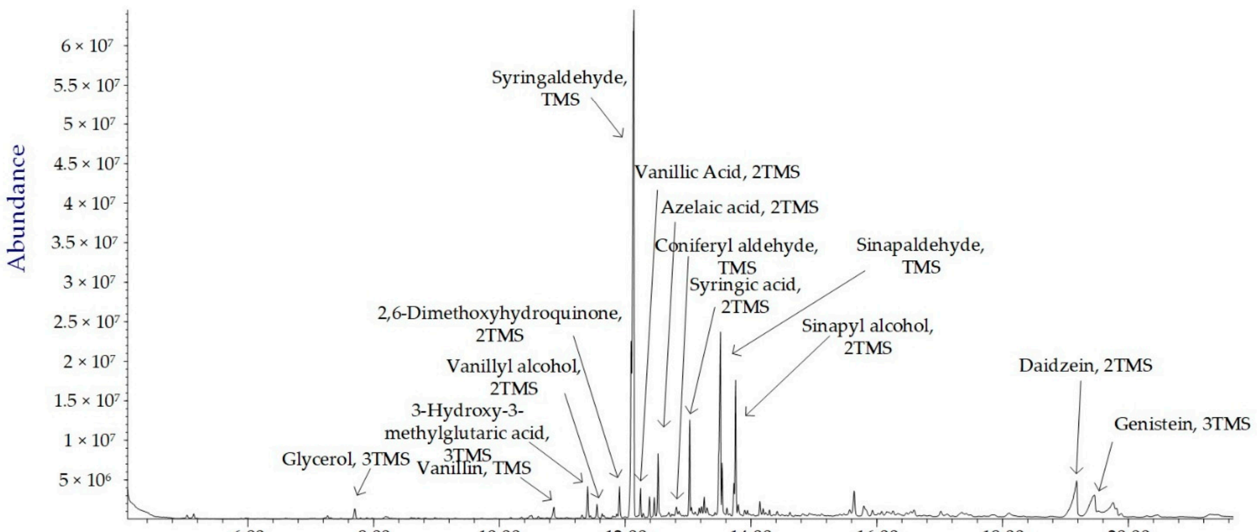

Figure S7. Annotated Total Ion Chromatograms (TICs) of crude extracts (*n*-hexane and dichloromethane), after derivatization with *N,O*-bis(trimethylsilyl)trifluoroacetamide (BSTFA), of roots of *Genista ulicina*.
